# Synthesis and Characterization of Eco-Efficient Alkali-Activated Composites with Self-Cleaning Properties for Sustainable Construction

**DOI:** 10.3390/molecules28166066

**Published:** 2023-08-15

**Authors:** Agnieszka Ślosarczyk, Izabela Klapiszewska, Patryk Jędrzejczak, Weronika Jędrzejczak, Łukasz Klapiszewski

**Affiliations:** 1 Institute of Building Engineering, Faculty of Civil and Transport Engineering, Poznan University of Technology, Piotrowo 3, PL-60965 Poznan, Poland; izabela.klapiszewska@put.poznan.pl (I.K.); weronika.jedrzejczak@poczta.onet.pl (W.J.); 2 Institute of Chemical Technology and Engineering, Faculty of Chemical Technology, Poznan University of Technology, Berdychowo 4, PL-60965 Poznan, Poland; patryk.jedrzejczak@doctorate.put.poznan.pl

**Keywords:** green technologies, alkali-activated materials, blast furnace slag, fly ash, titanium dioxide

## Abstract

In this research, we aimed to design an eco-efficient composite based on alkali-activated materials (AAMs) with self-cleaning properties for sustainable construction. Significant emphasis was placed on determining the role of the type of precursor, the amount of sodium silicate, and the addition of titanium dioxide on the rheological and mechanical properties of AAMs. An important aspect of the research was the modification of AAM with titanium dioxide to obtain the self-cleaning properties. Titanium dioxide, thanks to its photocatalytic properties, enables the reduction of organic pollutants and nitrogen oxides in the urban atmosphere and promotes the cleaning of material surfaces. Blast furnace slag (BFS) was used as the source material, which was then substituted in subsequent formulations with metakaolinite at 50% and fly ash and zeolite at 30%. The best-activated AAMs, in which blast furnace slag and its mixture with metakaolinite were used as precursors, achieved compressive strengths of 50 MPa. BFS mixtures with pozzolans were more difficult to polymerize, although their final strengths were still relatively high, in the range of 33–37 MPa. Adding titanium dioxide (T) improved the final strengths and slightly lowered the heat of hydration and spreading of the AAM mortars. The best self-cleaning properties were achieved with composites that comprised a mixture of blast furnace slag, fly ash, and 2% titanium dioxide.

## 1. Introduction

The construction industry is responsible for more than 40% of energy consumption; thus, the need to reduce the energy use during both the manufacturing of materials or components of building structures and for their maintenance is becoming increasingly clear [1,2]. Most structural building materials, e.g., steel, cement binders for concrete, and ceramics, are created via high-temperature processes, and due to the growth of the human population and the development of the global economy, the production of building materials will also increase in years to come [2,3,4,5]. This has led to an increase in the design and manufacture of new building materials based on waste or recycled materials, with the aim of reducing costs and energy consumption during production and, in accordance with the principles of the closed-loop economy, counteracting landfill waste [6,7,8].

One such solution is alkaline-activated materials, AAMs, which are created by activating typically aluminosilicate-rich post-industrial waste with sodium silicates, sodium, or potassium hydroxides, or a combination thereof [9,10,11,12,13]. The binding effect of the polymerization reaction can provide an alternative to cementitious binders and is more environmentally friendly due to its reduced emission of greenhouse gases such as CO_2_ and its lower global warming potential. In addition, concrete obtained with their contribution shows higher strength gains during the initial curing period, higher resistance to aggressive environments, and better resistance to temperature loads [14,15,16,17,18]. However, despite their many advantages, AAMs also have disadvantages, the most important of which appears to be the strong dependence of compressive strength on the type of precursor, the concentration and type of activator, and the temperature of the activation and curing process [19,20]. The best properties of AAMs are obtained when blast furnace slag BFS is activated with a mixture of sodium silicates and hydroxides. The polymerization reaction occurs with high efficiency even at room temperature, and the compressive strengths obtained reach values between 50 and 70 MPa, depending on the concentration of activators. It is much more difficult to activate metakaolinite (Mk), fly ash (FA), and other pozzolans such as natural zeolites (Ze), which, usually, in addition to high concentrations of hydroxides, also require an elevated activation temperature, usually between 60 and 90 °C, which in turn adversely affect the pro-environmental effect of these materials. Therefore, in order to achieve intermediate conditions for the synthesis of AAMs, mixtures of slag (SL) with various pozzolanic additives are becoming increasingly common [21,22,23,24,25,26].

Another way to accelerate the polymerization process and thicken the structure of AAMs is to modify the material at the nanometer level using oxide or carbon nanostructures. Research in this area has only recently emerged and still represents a significant knowledge gap in this field [27,28,29]. Thus far, nanometric titanium dioxide has been studied as a polymerization accelerator in AAMs, mainly in terms of its mechanical properties and ability to counteract shrinkage during setting. Studies have been carried out mainly for AAMs prepared from BFS, a mixture of BFS and Mk, and Mk alone, and it has been shown that nanometric titanium dioxide in amounts between 0.5 and 1% acts as a filler, thickening the structure of AAMs and thus contributing to lower shrinkage during the polymerization reaction, as well as increasing the flexural and compressive strength [30,31,32,33,34,35]. Nevertheless, titanium dioxide, thanks to its photocatalytic properties, also enables the reduction of organic pollutants and nitrogen oxides and promotes the cleaning of both the surface of building materials and the urban atmosphere. This phenomenon is known in the literature under the term self-cleaning properties and has been widely studied in the case of cement composites or natural stone, especially in public buildings or cultural heritage sites [36,37,38,39,40]. In contrast with these materials, the self-cleaning properties of titanium dioxide-induced AAMs composites have so far been rarely studied, with recent papers on the subject including Liano-Guerreo et al. and Coffetti et al. These works indicated that self-cleaning properties increase with increasing amounts of titanium dioxide, contrary to the mechanical parameters [33,34].

The literature review revealed that few papers describe the role of titanium dioxide in the mechanical and self-cleaning properties of AAMs made from BFS and Mk precursors and no articles describe other material combinations. Therefore, several objectives were formulated for the ongoing research presented in this article: (i) the intention was to determine the effect of the substitution of blast furnace slag with other additives in the form of fly ash, metakaolinite, and natural zeolite depending on the different contents of sodium silicate (SS) on the development of rheological, strength, and self-cleaning parameters of the AAMs obtained at ambient temperature, and (ii) to determine the effect of titanium dioxide on the above-mentioned parameters of AAMs synthesized from different precursors.

## 2. Results

**Figure 1:**
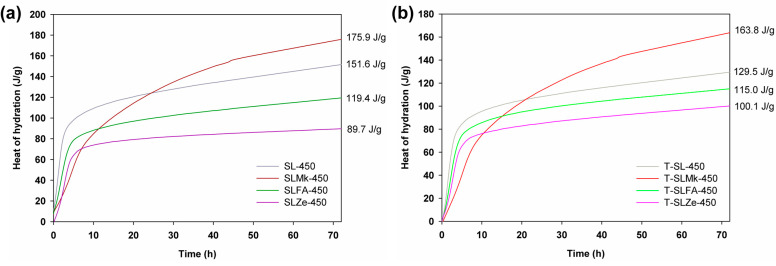
Hydration heat results for (**a**) references samples and (**b**) cement composites doped with TiO_2_ made of 450 g of SS.

**Figure 2:**
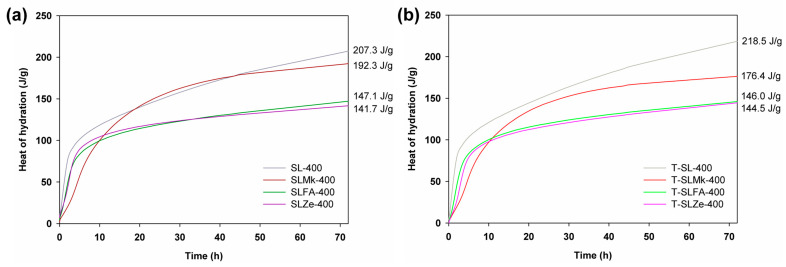
Hydration heat results for (**a**) references samples and (**b**) cement composites doped with TiO_2_ made of 400 g of SS.

Figure 1 and Figure 2 show the heat of hydration curves for the produced AAMs depending on the amount of activator, which was sodium silicate, and the type of precursor, which was blast furnace slag, a 50:50 mixture of blast furnace slag and metakaolinite, and a 70:30 mixture of blast furnace slag with silica fly ash and natural zeolite, respectively. It was determined that 450 g of sodium silicate was used in the first set of formulations shown in Figure 1, while in the second set, the amount of activator was reduced to 400 g and was supplemented with 50 g of water (see Figure 2). The ratio of activator and water to precursor was 1:1. For formulations with 450 g of activator, the highest heat of hydration was obtained for composites made from blast furnace slag, followed by slightly lower heat of hydration obtained for a mixture of slag with metakaolinite, fly ash, and zeolite. The released heat of hydration, which is a measure of the progress of the polymerization process, clearly confirmed that blast furnace slag and its mixture with metakaolinite were very easily activated at room temperature. The pozzolanic additives (here, fly ash and zeolite) were slightly less well activated under these conditions, which was reflected in a lowering of the heat of hydration and a much slower polymerization reaction. Similar correlations were obtained for formulations in which titanium dioxide was added at 2 wt.%, and with the exception of the formulation with zeolite, the addition of the oxide resulted in a slight reduction in the hydration heat released. In contrast, for formulas in which the amount of sodium silicate was reduced to 400 g and water was added, a significant increase in the heat of hydration was noted in all cases. Other correlations were similar to those for the higher amount of activator, with the exception of the recipe made from blast furnace slag, for which the addition of titanium dioxide accelerated the polymerization process and further increased the value of the released heat of hydration after 72 h of testing.

In turn, Table 1 shows the spreads of the produced AAMs and the density values of the obtained mortars. Mortars containing a higher SS content were characterized by a lower spread, while the addition of water at the expense of SS clearly improved the workability of the mortars, regardless of the type of precursor used. Smaller spreading values of 16–17 cm were recorded for the composite made from blast furnace slag alone and a mixture of BFS and zeolite. In other cases, the spread values ranged from 20–27 cm, obtaining the highest values for a mixture of slag and fly ash. Titanium dioxide slightly worsened mortar spreads, only in the case of mortar made on pure slag and 450 g of SS, and an improvement in the workability of the composite was noted. The densities of the produced AAMs were comparable in the range from 2.1 to 2.2 g/cm^3^.

**Figure 3:**
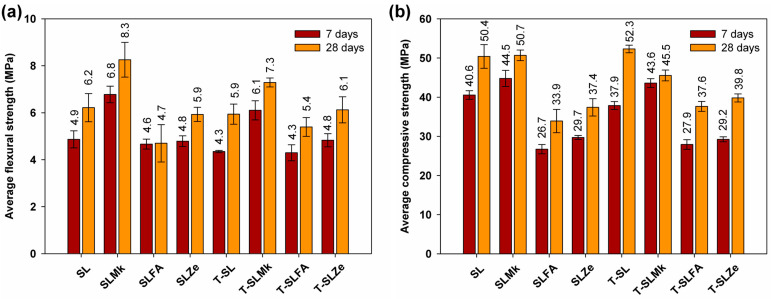
Mechanical strength test results for samples containing 450 g of waterglass: (**a**) average flexural strength and (**b**) average compressive strength.

**Figure 4:**
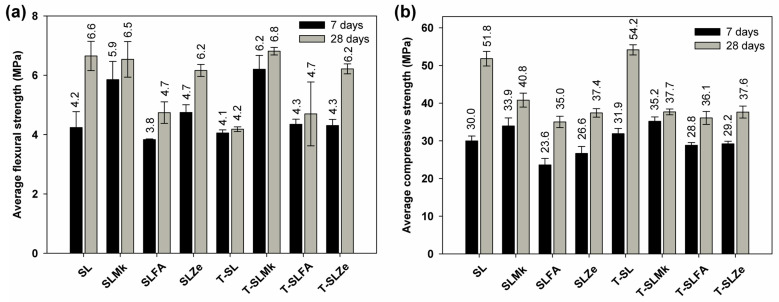
Mechanical strength test results for samples containing 400 g of waterglass: (**a**) average flexural strength and (**b**) average compressive strength.

Figure 3 and Figure 4 show the flexural and compressive strengths of composites made from 450 g SS and 400 g SS, which differed in terms of composition and the presence of titanium dioxide. The results of the flexural and compressive strengths reflect the results of the heat of hydration. Formulations containing blast furnace slag and a mixture of blast furnace slag and metakaolinite were most easily activated with sodium silicate. Artificial and natural pozzolans in the form of fly ash and zeolite were much more difficult to activate, which resulted in reduced flexural and compressive strength parameters. Flexural strength values ranged from 4.7 to 8.3 MPa, while compressive strengths ranged from 33.9 to 50.4 MPa. In all cases, adding titanium dioxide in the initial curing period of up to 7 days slightly reduced the strength parameters, or was an inert additive, which is consistent with the heat of hydration results. A positive effect of titanium dioxide on flexural strength, and especially on compressive strength, was noted only after 28 days of maturation; a slight increase in strength parameters was obtained, which indicates that the structure of AAMs was compacted by nanometric titanium dioxide. Reducing the amount of SS to 400 g and replacing it with water in the second configuration of the composites resulted in a decrease in mechanical parameters after both 7 and 28 days of maturation, except for the formulation based on pure blast furnace slag. In this case, an increase in flexural and compressive strength compared with the formulations made with 450 g SS was noted. This is due to the latent properties of BFS, which, although slow, reacts with water to form hydrated calcium aluminosilicates [41,42]. In other formulations in which BFS was substituted with metakaolinite and pozzolans, this increase was not observed, as these substances do not react with water, and the reduced amount of BFS was insufficient to gain significant benefits from its reaction with water. In these cases, the predominant reaction was the polymerization reaction of the precursors with SS, and, unfortunately, the lower SS content resulted in lower strength parameters of the AAMs.

**Figure 5:**
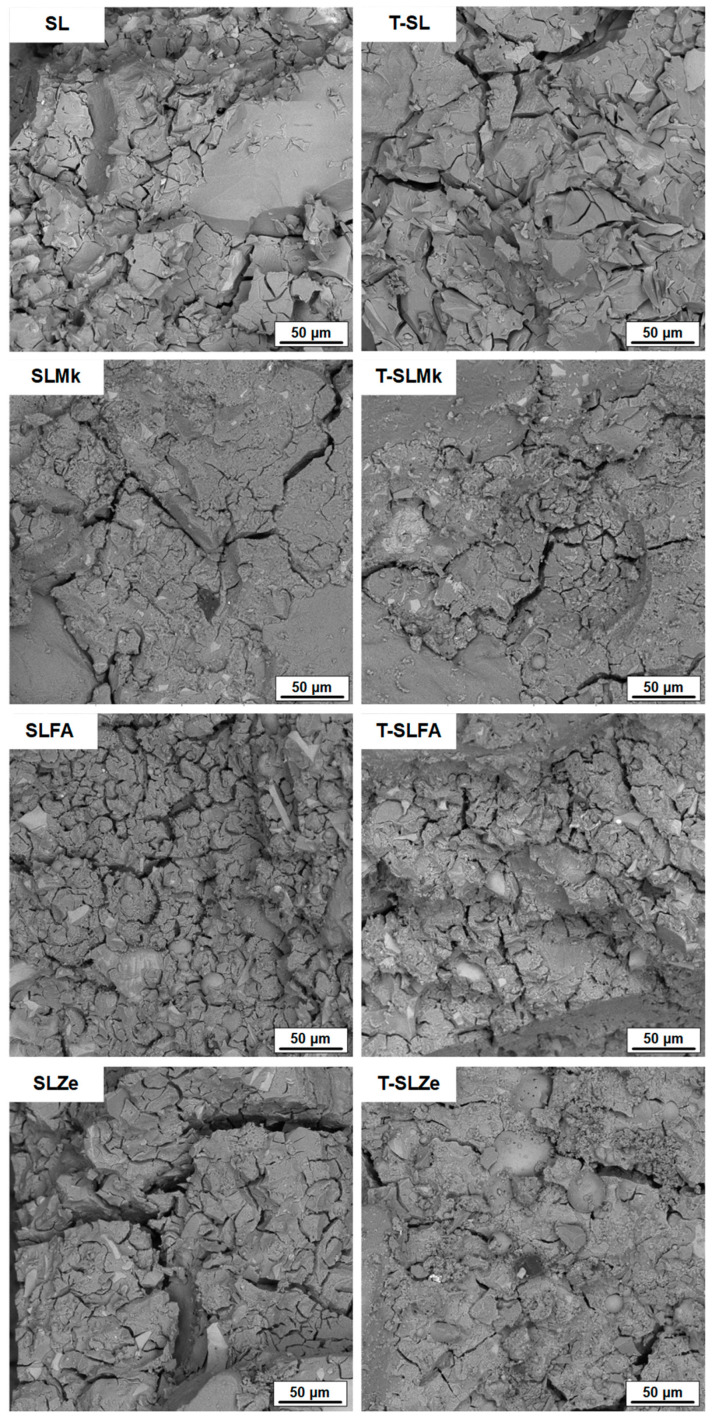
SEM images of samples containing 450 g of waterglass.

**Figure 6:**
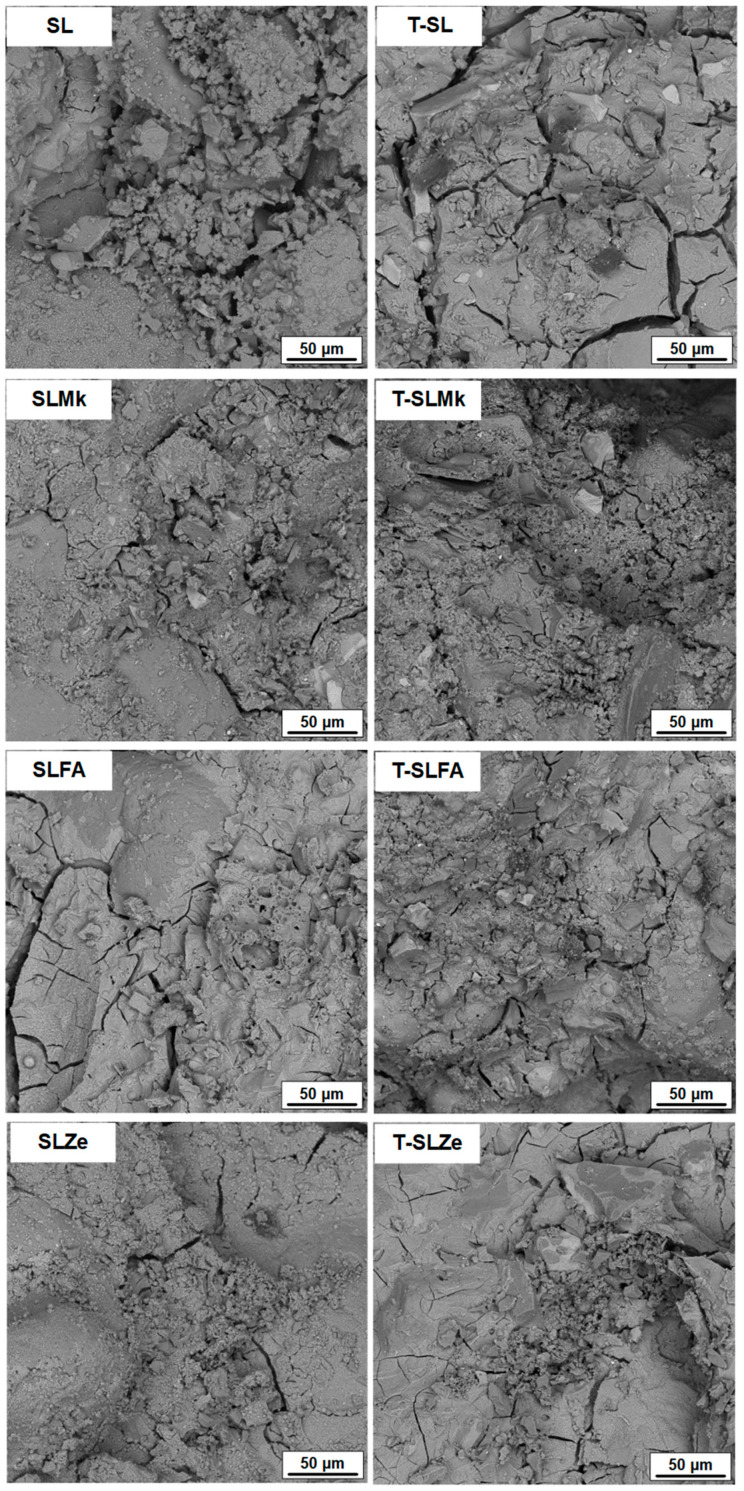
SEM images of samples containing 400 g of waterglass.

Figure 5 and Figure 6 show images of the microstructure of the fabricated AAMs, which confirm the results of the strength parameters. The most compact and homogeneous microstructures were obtained for composites made from blast furnace slag (SL) and from a mixture of blast furnace slag and metakaolinite (SLMk). For AAMs in which part of the slag was replaced by fly ash (SLFA) and zeolite (SLZe), the microstructure was more porous and fractured, and unreacted pozzolan grains were also visible. In turn, in all cases, the addition of water at the expense of SS (see Figure 6) resulted in a slight improvement in the homogeneity of the mortars, while no apparent change in microstructure was observed when titanium dioxide was added.

### Photocatalytic Assessment

**Figure 7:**
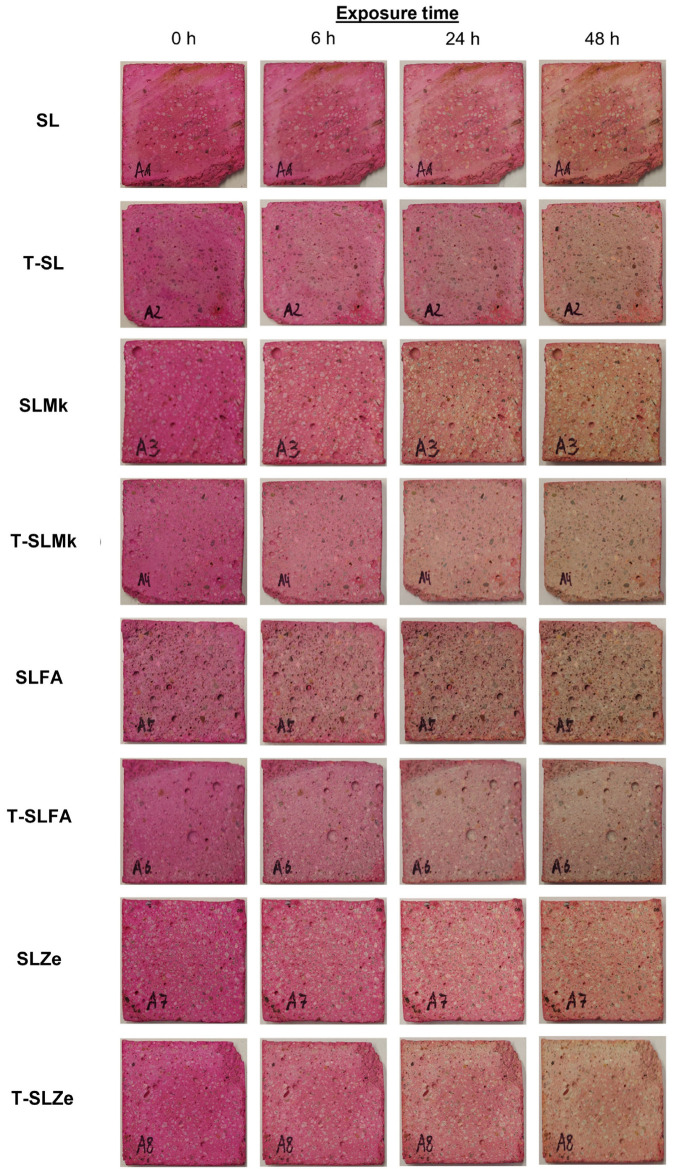
Photocatalytic assessment of geopolymer samples with 450 g of waterglass.

**Figure 8:**
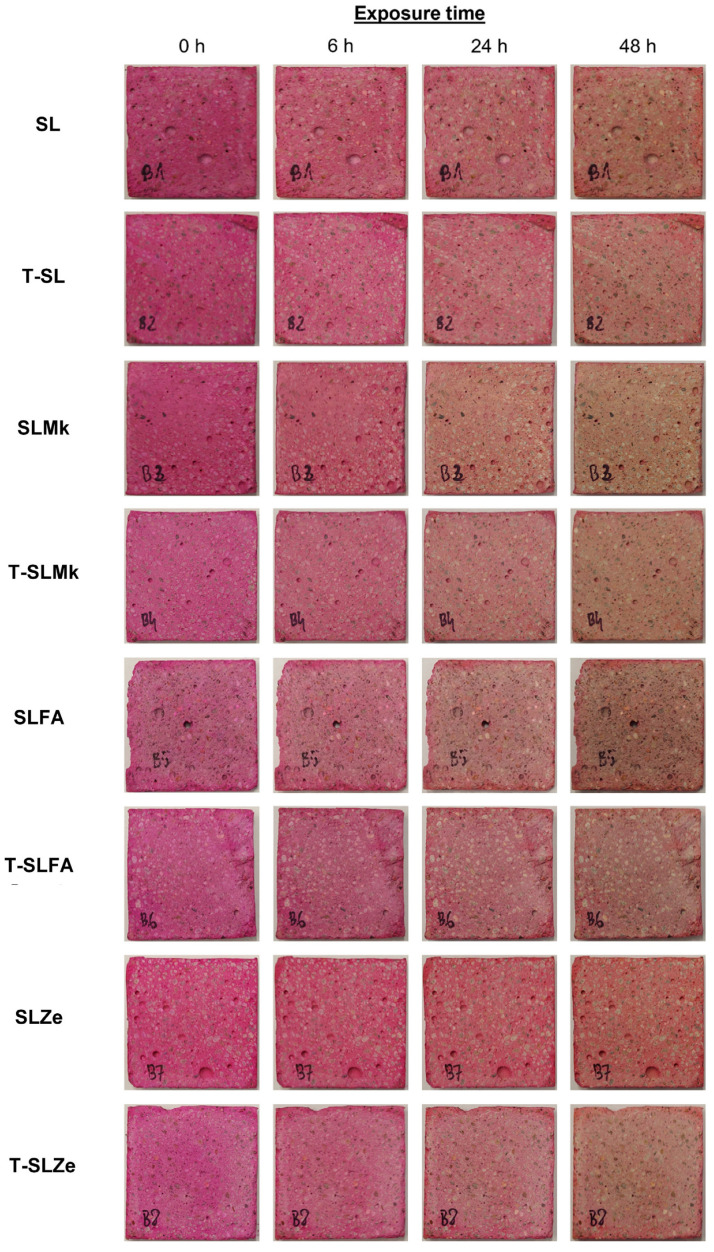
Photocatalytic assessment of geopolymer samples with 400 g of waterglass.

In order to evaluate the self-cleaning properties of AAMs, digital photos of the samples presented in Figure 7 and Figure 8 were analyzed. Nevertheless, there were significant differences between the individual samples. The AAMs to which titanium(IV) oxide nanoparticles were introduced showed stronger self-cleaning properties, which were particularly noticeable in SLMk-450 and SLMk-400 samples and their counterparts with the addition of TiO_2_. The worst self-cleaning properties, as evidenced by the lowest degree of discoloration of the AAM composite, were exhibited by samples with pure blast furnace slag and a mixture of BFS and zeolite SL-450, SLZe-450, SL-400, and SLZe-400. In these samples, after introducing additional TiO_2_ particles into the matrices, the discussed parameter improved. The T-SLFA-450 and T-SLMk-450 samples exhibited the best self-cleaning properties and almost complete discoloration after 48 h of irradiation.

## 3. Discussion

**Figure 9:**
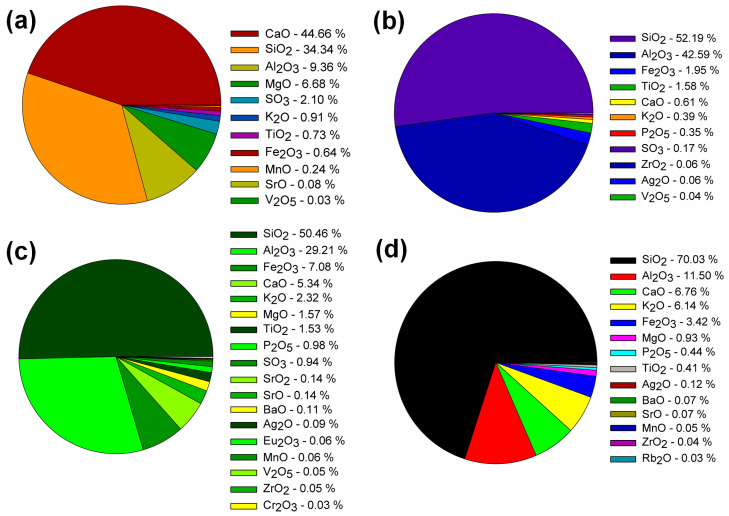
X-ray fluorescence (XRF) analysis of slag (**a**), metakaolinite (**b**), fly ash (**c**), and zeolite (**d**) used in this study.

In contrast with the results available in the literature for AAMs, this paper describes eco-efficient composites that differ in the type of precursor and contain titanium dioxide as an additive to impart self-cleaning properties to AAMs. Thus far, such research has been conducted to a limited extent and has been based mainly on blast furnace slag and metakaolinite matrices. Taking these two precursors into account, the research presented in this article has confirmed that both BFS and Mk activation with sodium silicate yields tough composites characterized by a high degree of polymerization and compacted structure. The high strength parameters are due to the presence of sodium aluminosilicates in the case of Mk, and interpenetrating networks of sodium and calcium aluminosilicates for the mixture of BFS and Mk, which was also confirmed in the work of other researchers [33]. The performance of AAM composites depending on the type of precursor is an issue of importance in view of the practical application of this material in construction and their wider use as an alternative to cementitious binder. Therefore, its performance has been the subject of many articles in recent years, including a paper by Bernal et al. comparing the performance of AAMs composites made on blast furnace slag with composites on ordinary Portland cement [43]. It was shown that at different binder percentages of 300, 400, and 500 kg/m^3^, AAM matrices showed comparable or higher mechanical properties than cementitious binders, as well as a lower porosity and absorbability, and thus higher resistance to external influences, such as chloride penetration. This trend was also confirmed in publications by Li et al. [44,45]. A higher performance was observed in AAMs composites using blast furnace slag and metakaolinite, which is consistent with our results. The higher lime contents associated with the presence of blast furnace slag and the Ca/(Al + Si) ratio resulted in an increased composite density and higher compressive strength. This is related to the formation of the C-S-H phase due to the activation of the blast furnace slag. On the other hand, the addition of fly ash at the expense of slag in amounts of 20, 30, and 40% resulted in an increase in slump and increased setting time, which in turn also generated a decrease in compressive strength. Pozzolans, fly ash, and zeolite are very difficult to activate via sodium silicate at room temperature. Hence, FA often requires the use of higher activation temperatures, to the order of 60–90 °C. In addition, activation with strong hydroxides or a mixture of silicates and hydroxides often occurs. Otherwise, the compressive strengths obtained are very low, making the structural use of AAMs impossible [46,47]. Zeolites undergo polymerization even more difficult than that of FA; for example, in the work of Nikolov et al., zeolites were activated with sodium silicate, sodium hydroxide, and sodium carbonate, and it was shown that the best parameters were obtained for activation with pure sodium silicate; nevertheless, the obtained compressive strengths were only a few MPa [42]. Thus, it is becoming increasingly common to combine pozzolanic precursors with blast furnace slag to avoid elevated temperatures and high concentrations of hydroxides, which affect the carbon footprint of AAM [19,22]. In our case, the matrices obtained in this way were characterized by very good mechanical parameters; the compressive strengths obtained when substituting BFS with fly ash and zeolite ranged from 34 to 37 MPa. In addition, the use of titanium dioxide in the amount of 2% by weight improved the strength parameters in all configurations. Similar correlations were also obtained by the authors of [30,31,33,34], in which titanium dioxide (in pure form or as a composite with graphene added in amounts from 0.5 to 1%) improved the compressive strength of AAMs. Larger amounts worsened the mechanical parameters, but positively influenced the self-cleaning properties, which was confirmed in the papers of Liano-Guerreo et al. [33] and Coffetti et al. [34]. In the presented paper, a visual evaluation of the samples subjected to ultraviolet (UV) irradiation in the presence of the Rhodamine B indicator showed that adding titanium dioxide to AAMs imparted self-cleaning properties, with the effectiveness of titanium dioxide depending on the type of precursor used. Good self-cleaning properties were noted for the matrix with BFS and Mk, which obtained the highest strength parameters, while the most favorable ones were recorded for the matrix containing BFS and fly ash. This is probably due to the composition of metakaolinite and fly ash, shown in Figure 9, as they contain titanium dioxide in an amount greater than 1.5 wt.% and enhanced the photocatalytic effect of nanometric TiO_2_. AAMs obtained from BFS alone and from a mixture of BFS and zeolite showed the worst self-cleaning properties.

## 4. Materials and Methods

Slag (SL) (Lafarge Cement S.A., Warszawa, Poland), metakaolinite (Mk) ASTRA MK-40 (Astra, Straszyn, Poland), zeolite (Ze) ASTRA Z-50 (Astra, Straszyn, Poland), and fly ash (FA) (Elektrownia Opole, Opole, Poland) were used in the study. The oxide composition of all binders is presented in Figure 9. The aggregate was quartz sand (Kwarcmix, Tomaszów Mazowiecki, Poland) with a grain size of 0–2 mm. The additive used in the composite was titanium (IV) oxide, anatase form (T), CAS number: 1317-70-0 (Sigma-Aldrich, Saint Louis, MO, USA). The composition and markings of the produced samples of the geopolymer composites are summarized in Table 2.

**Figure 10:**
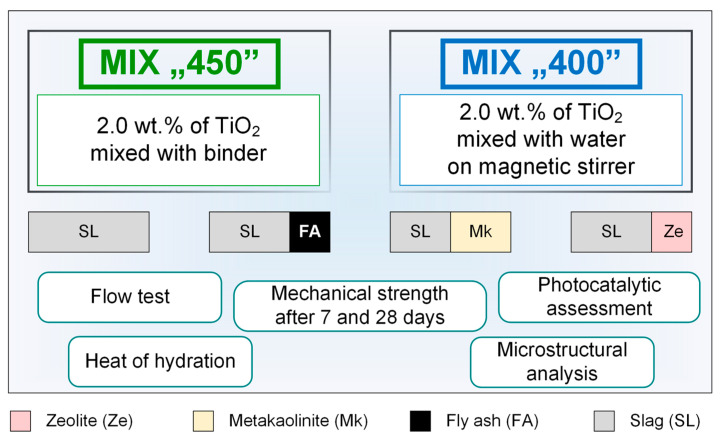
Schematic representation of the performed analysis.

The schematic diagram of the tests and analyses performed is shown in Figure 10. The composite mixing procedure was carried out in accordance with the standard procedure for producing cement composites according to PN-EN 196-1, which was described in detail in an earlier work [48,49]. For the samples prepared using waterglass alone, the TiO_2_ admixture was introduced directly with the dry ingredients into the mixer bowl, while in the case of composites containing the addition of water, it was first mixed with water on a magnetic stirrer (approx. 5 min), and then in the form of a suspension poured into the mixing bowl together with the appropriate amount of waterglass. Mixing was performed using an automatic mixer; bar samples in molds (dimension 40 mm × 40 mm × 160 mm) were condensed with 60 strokes. After demolding, the samples were stored in water prior to mechanical testing. The flow test was carried out on a shaking table, according to the PN-EN 1015-3 standard procedure; a freshly prepared geopolymer mortar was applied in two layers in a ring on the shaking table, then the ring was removed and the mortar was subjected to 15 shocks. The resulting flow was measured in two orthogonal directions.

The hydration heat test was carried out using a TESTING semi-adiabatic calorimeter (TESTING Bluhm and Feuerherdt GmbH, Berlin, Germany), according to the PN-EN 196-9 standard procedure. The test lasted 72 h. The assessment of the mechanical properties after 7 and 28 days was conducted using a Matest ServoPlus Evolution testing machine (Matest SpA, Treviolo, BG, Italy) equipped with two channels—one for flexural strength testing and the other for compression. The test was carried out according to PN-EN 196-1, where six samples with dimensions of 40 mm × 40 mm × 160 mm are placed in the first channel, axially, on two rollers placed at a distance of 100 mm. The breaking force is applied in increments of 50 ± 10 N/s until the sample broke. In the second channel, the 12 halves of the beams obtained after the flexural test one by one were successively placed between two square plates. The load was increased in steps of 2.4 ± 0.2 kN/s until the sample failed. The conducted analysis of the microstructure of geopolymer composites was carried out using a Tescan VEGA3 scanning electron microscope (TESCAN Orsay Holding a.s., Brno, Czech Republic).

To determine the light-induced self-cleaning properties of the prepared geopolymer composites, a test of the photocatalytic degradation of a model organic pollutant, i.e., Rhodamine B, against ultraviolet radiation as a light source was carried out. Therefore, composites samples measuring 40 mm × 40 mm × 5 mm were immersed in an aqueous solution of Rhodamine B (Sigma Aldrich, Saint Louis, MO, USA) at a concentration of 500 mg/L for 1 h. The geopolymer samples were then held at 90 °C for 24 h. Subsequently, the self-cleaning properties test began, during which the composites were exposed to ultraviolet radiation with a wavelength of 395 nm, which was emitted by a 50 W UV-LED lamp (Bridgelux Inc., Fremont, CA, USA). Then, after 6, 24, and 48 h, a visual assessment of the photocatalytic degradation of Rhodamine B was performed. As a result of the self-cleaning properties of the composites, it was predicted that the color typical for this pigment would disappear.

## 5. Conclusions

Based on this research, the following conclusions can be drawn:

- The degree of activation of AAMs depends on the amount of sodium silicate and the type of precursor. The best strength parameters were obtained for BFS and a mixture of BFS and Mk. Water added at the expense of sodium silicate promoted the hydration of blast furnace slag, and only for a matrix made on slag alone were the strength gains recorded. Matrices made of BFS and natural and artificial pozzolans obtained lower strengths, but, nevertheless, the compressive strength values were in the 34–37 MPa range.
- Titanium dioxide added at 2 wt.% caused strength gains, but only at a later maturation time. At the beginning of maturation, this retarded the setting, resulting in a reduction in the heat of hydration, and it slightly reduced the workability of mortars and imparted self-cleaning properties to AAMs.
- Self-cleaning properties are dependent on the type of precursor. Metakaolinite and fly ash promoted and enhanced the photocatalytic effect of nanometric titanium dioxide.
- The designed AAM composites characterized by lower hydration heat and self-cleaning properties may provide a pro-environmental alternative to cementitious binder for green technologies.
- It is worth noting that producing composites with the addition of titanium dioxide particles will be less cost-effective compared with pure composites. However, the improvement in mechanical properties and new features, such as self-cleaning properties, make TiO_2_-doped composites an attractive material. To enhance the cost-effectiveness of using TiO_2_ particles as additives in composites, one can consider the use of waste products.

## Figures and Tables

**Table 1:** Flow test results and density of cement composites.

Sample	“450”		“400”	
	Slump Test (cm)	Density (g/cm^3^)	Slump Test (cm)	Density (g/cm^3^)
SL	17.3 ± 0.3	2.19 ± 0.02	24.3 ± 0.3	2.17 ± 0.03
SLMk	20.8 ± 0.3	2.18 ± 0.02	26.3 ± 0.3	2.16 ± 0.02
SLFA	22.8 ± 0.3	2.09 ± 0.04	27.3 ± 0.3	2.12 ± 0.01
SLZe	16.5 ± 0.0	2.19 ± 0.03	19.3 ± 0.3	2.19 ± 0.02
T-SL	23.3 ± 0.3	2.26 ± 0.02	23.0 ± 0.5	2.23 ± 0.00
T-SLMk	19.0 ± 0.0	2.14 ± 0.02	25.8 ± 0.0	2.13 ± 0.00
T-SLFA	19.5 ± 0.5	2.17 ± 0.01	27.5 ± 0.5	2.14 ± 0.02
T-SLZe	16.0 ± 0.0	2.20 ± 0.01	15.5 ± 0.5	2.15 ± 0.02

**Table 2:** Sample composition presented in this study.

Sample	Components (g)							
	Slag	Metakaolinite	Fly Ash	Zeolite	Glass Water	Water	Aggregate	TiO_2_
SL-450	450	-	-	-	450	-	1350	-
SLMk-450	225	225	-	-				
SLFA-450	315	-	135	-				
SLZe-450	315	-	-	135				
T-SL-450	450	-	-	-				9
T-SLMk-450	225	225	-	-				
T-SLFA-450	315	-	135	-				
T-SLZe-450	315	-	-	135				
SL-400	450	-	-	-	400	50	1350	-
SLMk-400	225	225	-	-				
SLFA-400	315	-	135	-				
SLZe-400	315	-	-	135				
T-SL-400	450	-	-	-				9
T-SLMk-400	225	225	-	-				
T-SLFA-400	315	-	135	-				
T-SLZe-400	315	-	-	135				

## Data Availability

Data will be made available upon request.

## Sample Availability

Samples of the compounds are available from the authors.

## References

[1] Pérez-Lombard L., Ortiz J., Pout C. (2008). A review on buildings energy consumption information. Energy Build..

[2] Monteiro H., Cruz P.L., Moura B. (2022). Integrated environmental and economic life cycle assessment of improvement strategies for a ceramic industry. J. Clean. Prod..

[3] Olmez G.M., Dilek F.B., Karanfil T., Yetis U. (2016). The environmental impacts of iron and steel industry: A life cycle assessment study. J. Clean. Prod..

[4] Van Damme H. (2018). Concrete material science: Past, present, and future innovations. Cem. Concr. Res..

[5] Almutairi A.L., Tayeh B.A., Adesina A., Isleem H.F., Zeyad A.M. (2021). Potential applications of geopolymer concrete in construction: A review. Case Stud. Constr. Mater..

[6] Miller S.A., John V.M., Pacca S.A., Horvath A. (2018). Carbon dioxide reduction potential in the global cement industry by 2050. Cem. Concr. Res..

[7] Schneider M. (2019). The cement industry on the way to a low-carbon future. Cem. Concr. Res..

[8] Scrivener K.L., John V.M., Gartner E.M. (2018). Eco-efficient cements: Potential economically viable solutions for a low-CO_2_ cement-based materials industry. Cem. Concr. Res..

[9] Ślosarczyk A., Fořt J., Klapiszewska I., Thomas M., Klapiszewski Ł., Černý R. (2023). A literature review of the latest trends and perspectives regarding alkali-activated materials in terms of sustainable development. J. Mater. Res. Technol..

[10] Shi C., Fernández Jiménez A., Palomo A. (2011). New cements for the 21st century: The pursuit of an alternative to Portland cement. Cem. Concr. Res..

[11] Gao X., Yao X., Wang C., Geng C., Yang T. (2022). Properties and microstructure of eco-friendly alkali-activated slag cements under hydrothermal conditions relevant to well cementing applications. Constr. Build. Mater..

[12] Zhang P., Zheng Y., Wang K., Zhang J. (2018). A review on properties of fresh and hardened geopolymer mortar. Compos. B Eng..

[13] Awoyera P., Adesina A. (2019). A critical review on application of alkali activated slag as a sustainable composite binder. Case Stud. Constr. Mater..

[14] Nodehi M., Ozbakkaloglu T., Gholampour A., Mohammed T., Shi X. (2022). The effect of curing regimes on physico-mechanical, microstructural and durability properties of alkali-activated materials: A review. Constr. Build. Mater..

[15] de Oliveira L.B., de Azevedo A.R.G., Marvila M.T., Pereira E.C., Fediuk R., Vieira C.M.F. (2022). Durability of geopolymers with industrial waste. Case Stud. Constr. Mater..

[16] Tahir M.F.M., Al Bakri Abdullah M.M., Abd Rahim S.Z., Hasan M.R.M., Sandu A.V., Vizureanu P., Ghazali C.M.R., Kadir A.A. (2022). Mechanical and Durability Analysis of Fly Ash Based Geopolymer with Various Compositions for Rigid Pavement Applications. Materials.

[17] Thunuguntla C.S., Rao T.D.G. (2018). Effect of mix design parameters on mechanical and durability properties of alkali activated slag concrete. Constr. Build. Mater..

[18] Athira V.S., Bahurudeen A., Saljas M., Jayachandran K. (2021). Influence of different curing methods on mechanical and durability properties of alkali activated binders. Constr. Build. Mater..

[19] Sun B., Ye G., de Schutter G. (2022). A review: Reaction mechanism and strength of slag and fly ash-based alkali-activated materials. Constr. Build. Mater..

[20] Lyu B.-C., Ding C., Guo L.-P., Chen B., Wang A.-G. (2021). Basic performances and potential research problems of strain hardening geopolymer composites: A critical review. Constr. Build. Mater..

[21] Phoo-ngernkham T., Maegawa A., Mishima N., Hatanaka S., Chindaprasirt P. (2015). Effects of sodium hydroxide and sodium silicate solutions on compressive and shear bond strengths of FA–GBFS geopolymer. Constr. Build. Mater..

[22] Humad A.M., Kothari A., Provis J.L., Cwirzen A. (2019). The Effect of Blast Furnace Slag/Fly Ash Ratio on Setting, Strength, and Shrinkage of Alkali-Activated Pastes and Concretes. Front. Mater..

[23] Abdila S.R., Al Bakri Abdullah M.M., Ahmad R., Nergis D.D.B., Abd Rahim S.Z., Omar M.F., Sandu A.V., Vizureanu P., Syafwandi (2022). Potential of Soil Stabilization Using Ground Granulated Blast Furnace Slag (GGBFS) and Fly Ash via Geopolymerization Method: A Review. Materials.

[24] Mounika G., Ramesh B., Kalyana Rama J.S. (2020). Experimental investigation on physical and mechanical properties of alkali activated concrete using industrial and agro waste. Mater. Today Proc..

[25] Chi M., Huang R. (2013). Binding mechanism and properties of alkali-activated fly ash/slag mortars. Constr. Build. Mater..

[26] Longo F., Lassandro P., Moshiri A., Phatak T., Aiello M.A., Krakowiak K.J. (2020). Lightweight geopolymer-based mortars for the structural and energy retrofit of buildings. Energy Build..

[27] Jindal B.B., Sharma R. (2020). The effect of nanomaterials on properties of geopolymers derived from industrial by-products: A state-of-the-art review. Constr. Build. Mater..

[28] Shilar F.A., Ganachari S.V., Patil V.B. (2022). Advancement of nano-based construction materials-A review. Constr. Build. Mater..

[29] Raza A., El Ouni M.H., Azab M., Ali K., Haider H., Rashedi A. (2022). A scientometric review on mechanical and durability performance of geopolymer Paste: Effect of various raw materials. Constr. Build. Mater..

[30] Yang L.Y., Jia Z.J., Zhang Y.M., Dai J.G. (2015). Effects of nano-TiO_2_ on strength, shrinkage and microstructure of alkali activated slag pastes. Cem. Concr. Compos..

[31] Zhang S.-L., Qi X.-Q., Guo S.-Y., Ren J., Chen J.-Z., Chi B., Wang X.-C. (2021). Effect of a novel hybrid TiO_2_-graphene composite on enhancing mechanical and durability characteristics of alkali-activated slag mortar. Constr. Build. Mater..

[32] Duan P., Yan C., Luo W., Zhou W. (2016). Effects of adding nano-TiO_2_ on compressive strength, drying shrinkage, carbonation and microstructure of fluidized bed fly ash based geopolymer paste. Constr. Build. Mater..

[33] Llano-Guerrero E.A., Gómez-Zamorano L.Y., Jiménez-Relinque E. (2020). Effect of the addition of TiO_2_ nanoparticles in alkali-activated materials. Constr. Build. Mater..

[34] Coffetti D., Crotti E., Coppola L. (2023). Long-term properties of self-cleaning alkali-activated slag-based mortars with titanium dioxide nanoparticles. Constr. Build. Mater..

[35] El-Kattan I.M., Saif M.S., El-Hariri M.O.R., Elgandy A.H., Pérez-Villarejo L., Eliche-Quesada D. (2023). Assessing the individual impact of magnesia and titania nano- particles on the performance of alkali-activated slag mortars. Constr. Build. Mater..

[36] La Russa M.F., Rovella N., Alvarez de Buergo M., Belfiore C.M., Pezzino A., Crisci G.M., Ruffolo S.A. (2016). Nano-TiO_2_ coatings for cultural heritage protection: The role of the binder on hydrophobic and self-cleaning efficacy. Prog. Org. Coat..

[37] Munafò P., Goffredo G.B., Quagliarini E. (2015). TiO_2_-based nanocoatings for preserving architectural stone surfaces: An overview. Constr. Build. Mater..

[38] Janczarek M., Klapiszewski Ł., Jędrzejczak P., Klapiszewska I., Ślosarczyk A., Jesionowski T. (2022). Progress of functionalized TiO_2_-based nanomaterials in the construction industry: A comprehensive review. Chem. Eng. J..

[39] Dikkar H., Kapre V., Diwan A., Sekar S.K. (2021). Titanium dioxide as a photocatalyst to create self-cleaning concrete. Mater. Today Proc..

[40] Sikora P., Horszczaruk E., Rucinska T. (2015). The Effect of Nanosilica and Titanium Dioxide on the Mechanical and Self-Cleaning Properties of Waste-Glass Cement Mortar. Procedia Eng..

[41] Amran M., Murali G., Khalid N.H.A., Fediuk R., Ozbakkaloglu T., Lee Y.H., Haruna S., Lee Y.Y. (2021). Slag uses in making an ecofriendly and sustainable concrete: A review. Constr. Build. Mater..

[42] Nikolov A., Rostovsky I., Nugteren H. (2017). Geopolymer materials based on natural zeolite. Case Stud. Constr. Mater..

[43] Bernal S.A., de Gutiérrez R.M., Pedraza A.L., Provis J.L., Rodriguez E.D., Delvasto S. (2011). Effect of binder content on the performance of alkali-activated slag concretes. Cem. Concr. Res..

[44] Li N., Shi C., Wang Q., Zhang Z., Ou Z. (2017). Composition design and performance of alkali-activated cements. Mater. Struct..

[45] Li N., Shi C., Zhang Z., Zhu D., Hwang H.-J., Zhu Y., Sun T. (2018). A mixture proportioning method for the development of performance-based alkali-activated slag-based concrete. Cem. Concr. Compos..

[46] Salas D.A., Ramirez A.D., Ulloa N., Baykara H., Boero A.J. (2018). Life cycle assessment of geopolymer concrete. Constr. Build. Mater..

[47] Alhassan M., Alkhawaldeh A., Betoush N., Alkhawaldeh M., Huseien G.F., Amaireh L., Elrefae A. (2023). Life Cycle Assessment of the Sustainability of Alkali-Activated Binders. Biomimetics.

[48] Klapiszewska I., Ławniczak Ł., Balicki S., Gapiński B., Wieczorowski M., Wilk K.A., Jesionowski T., Klapiszewski Ł., Ślosarczyk A. (2023). Influence of zinc oxide particles dispersion on the functional and antimicrobial properties of cementitious composites. J. Mater. Res. Technol..

[49] Klapiszewska I., Parus A., Ławniczak Ł., Jesionowski T., Klapiszewski Ł., Ślosarczyk A. (2021). Production of antibacterial cement composites containing ZnO/lignin and ZnO–SiO_2_/lignin hybrid admixtures. Cem. Concr. Compos..

